# Estimating the Risk of ABO Hemolytic Disease of the Newborn in Lagos

**DOI:** 10.1155/2015/560738

**Published:** 2015-09-17

**Authors:** Alani Sulaimon Akanmu, Olufemi Abiola Oyedeji, Titilope Adenike Adeyemo, Ann Abiola Ogbenna

**Affiliations:** Department of Hematology & Blood Transfusion, Faculty of Clinical Sciences, College of Medicine, University of Lagos, PMB 12003, Lagos, Nigeria

## Abstract

*Background*. ABO hemolytic disease of the newborn is the most common hemolytic consequence of maternofetal blood group incompatibility restricted mostly to non-group-O babies of group O mothers with immune anti-A or anti-B antibodies. *Aim*. We estimated the risk of ABO HDN with view to determining need for routine screening for ABO incompatibility between mother and fetus. *Materials and Methods*. Prevalence of ABO blood group phenotypes in blood donors at the donor clinic of the Lagos University Teaching Hospital and arithmetic methods were used to determine population prevalence of ABO genes. We then estimated proportion of pregnancies of group O mothers carrying a non-group-O baby and the risk that maternofetal ABO incompatibility will cause clinical ABO HDN. *Results*. Blood from 9138 donors was ABO typed. 54.3%, 23%, 19.4%, and 3.3% were blood groups O, A, B, and AB, respectively. Calculated gene frequencies were 0.1416, 0.1209, and 0.7375 for A, B, and O genes, respectively. It was estimated that 14.3% of deliveries will result in a blood group O woman giving birth to a child who is non-group-O. Approximately 4.3% of deliveries are likely to suffer ABO HDN with 2.7% prone to suffer from moderately severe to severe hemolysis.

## 1. Introduction

ABO hemolytic disease of the newborn (ABO HDN) is the most common maternofetal blood group incompatibility. Unlike the rhesus disease, it is usually a problem of the neonate rather than the fetus. ABO HDN is restricted almost entirely to group A or B babies born to group O mothers with immune anti-A or anti-B antibodies.

ABO HDN is caused by IgG (immune) maternal antibodies which have the ability to cross the placental barrier. A high titre of these immune antibodies may not present with adverse effects in utero as A and B antigens are present on cells of all other tissues and body fluid and not only on red cells. The presence of these antigens helps to protect the incompatible fetal red cells by neutralizing the transferred maternal antibody with small amounts of antibody reacting directly with the fetal red cells [1]. The red cells which are sensitized by the antibodies are destroyed by macrophages in the fetal spleen with consequent hyperbilirubinaemia [2].

ABO-HDN in literature is described as a condition having a very low incidence in the population and characterized by a benign evolution because of a mild degree of hemolysis [3, 4]. Anaemia is rare with the main clinical problem being jaundice. Severe hemolysis and anaemia requiring exchange blood transfusion have however been reported [5]. Early detection and treatment of neonatal hyperbilirubinaemia is important in prevention of bilirubin-induced encephalopathy in the affected children [6].

The above statements, however, are not valid for all populations. Studies have revealed that statistically, mother and infant are ABO-incompatible in one of every five pregnancies among Caucasians [7, 8]. The incidence of ABO HDN in the United Kingdom is about 2% of all births, but severe hemolytic disease occurs in only 0.03% of births [9]. The incidence of ABO HDN in Blacks [10] is said to be higher than in Caucasians [11–13]. This is due to the higher prevalence and titres of immune anti-A and anti-B antibodies in the Black population [14–18].

Routine antenatal antibody screening tests (indirect Coombs test) do not routinely include screening for ABO HDN. Diagnosis is usually made by investigation of a newborn baby who has developed jaundice during the first day of life. Routine screening for ABO incompatibility between mother and fetus is not performed and according to Han et al. it is not cost effective to routinely screen for ABO incompatibility in the Asian population [19].

The prevalence of immune anti-A and anti-B antibodies and the population and gene frequencies of the various ABO blood groups are useful in predicting an estimate of children born by blood group O women married to non-group-O husbands who are at risk of developing ABO HDN.

This study aims at estimating the risk of ABO HDN in our population with a view to determining whether there is the need for routine screening since the incidence of ABO HDN is expected to be higher in Blacks.

## 2. Subjects and Methods

We determined the prevalence of ABO blood types among 9138 blood donors at Lagos University Teaching Hospital by collating the data of all donors over a one-year period. Arithmetical methods based on Hardy Weinberg equilibrium were then used to determine population prevalence of different ABO genes using the phenotype data obtained from ABO typing of blood donors (Appendix A).

The population prevalence of hemolysins in Lagos [20] in combination with the obtained ABO gene frequencies was used to calculate the likelihood of scenario where a non-blood-group-O baby will be born to a blood group O mother with immune ABO antibodies (Appendix B). These were then used to arrive at an estimate of the incidence of ABO HDN within the population.

## 3. Results

A total of 9138 blood donors were ABO blood group typed. 4962 (54.3%) were blood group O. Blood group A was slightly more prevalent (23.0%) than blood group B (19.4%). AB blood group constituted only 3.3% (Table 1).

The calculated population prevalence of the A, B, and O genes in Lagos is 0.1416, 0.1209, and 0.7375, respectively (details of calculations are presented in Appendix A).

The calculated probability of a blood group O woman giving birth to a child who is non-group-O phenotype is 14.3% of deliveries in Lagos (details of calculations are presented in Appendix B).

With a prevalence of anti-A and anti-B hemolysins in blood group O individuals of 30.3% and with 18.6% of blood group O donors having significant visual titres, approximately 4.3% of deliveries (30.3% of 14.3% deliveries) are likely to suffer ABO HDN with 2.7% of deliveries (18.6% of 14.3% deliveries) prone to suffer from moderately severe to severe hemolysis.

## 4. Discussion

The incidence of severe neonatal jaundice within the first few hours of life (bilirubin above 10 mg/100 mL) is fairly common with a significant number requiring exchange transfusions. However, the number of cases that are due to hemolysis from ABO incompatibility between mother and fetus have not yet been established. A 1 in 5 chance of ABO incompatibility between fetal red cells and maternal serum exists but the incidence of ABO HDN elsewhere is said to be uncommon occurring in 2% of all births [5, 9]. Race has however been shown to have an effect on the incidence and severity of ABO HDN with a higher incidence and severity being observed among Blacks [10] and Latin Americans [7]. Thus we can expect the incidence and severity of ABO HDN to be higher in Nigeria.

Blood group O individuals were 54.3% of the donor population which is consistent with those found in an earlier population study by Ahmed et al. in Lagos State [21]. As it can be assumed that sex factor has no influence on ABO gene inheritance and blood group distribution, then 54.3% of females in Lagos can be assumed to be blood group O.

From results obtained in this study using the gene frequencies of the ABO blood group antigens and then calculating the probabilities of a blood group O woman marrying a non-group-O man and having a non-group-O child, the incidence of ABO incompatible pregnancies in the population with the mother being blood group O is 14.3%. This is similar to results obtained by Cariani et al. [7] in Venezuela and what has been reported for anglosaxon countries [22]. This is due to the similar frequency of blood group O in the studied populations.

Anti-A and anti-B hemolysins prevalence in the study population is 30.3% with 18.6% having significant visual titres of 8 and above [20]. From calculations, 4.3% of deliveries in Lagos (30.3% of 14.3% deliveries) are likely to suffer ABO HDN. This is consistent with reports which have found that the incidence of ABO HDN is higher in Blacks than in Caucasians [11–13] and is double that of the figures obtained for the United Kingdom [9]. This higher finding can be explained by the higher prevalence of hemolysins in Black population. Visual titres of hemolysins of 8 and above have been associated with significant in vivo hemolysis [17, 23]. Assuming those with significant visual titre as being capable of leading to severe ABO HDN, potentially 2.7% of deliveries (18.6% of 14.3% deliveries) will have moderately severe to severe ABO HDN. This finding is considerably higher than in the United Kingdom where severe hemolytic disease occurs in only 0.03% of births [9].

Routine screening for ABO incompatibility is presently not performed in Lagos University Teaching Hospital with most babies discharged as soon as possible after delivery. Also, there is no test that is of high predictive value for severe HDN. It can however be suggested that hemolysis from ABO HDN can be more severe amongst Nigerian neonates whose mothers tend to have higher prevalence and titres of immune anti-A and anti-B antibodies from several studies [14–18]. Thus, there may be a case for routine screening for immune antibodies in pregnant blood group O women to monitor fetuses that may be at risk.

As routine testing for hemolysins is not performed in this environment, blood group O women with suspected high immune antibody titre or with a history of ABO incompatibility in a previous pregnancy may also require monitoring of their neonates.

## 5. Conclusion

The estimated risk of ABO HDN among non-group-O offspring of blood group O women is 4.3% of all deliveries in Lagos University Teaching Hospital. 2.7% of babies ABO incompatible with their mothers are at risk of moderately severe to severe HDN. With this finding, it may not be cost effective to routinely screen for ABO HDN. However, best practices for detecting neonatal jaundice need to be put in place and, if severe neonatal jaundice occurs in a setting of ABO incompatibility, intravenous immunoglobulin which usually avoids the invasive procedure of an exchange transfusion should be considered.

## Figures and Tables

**Figure 1:**
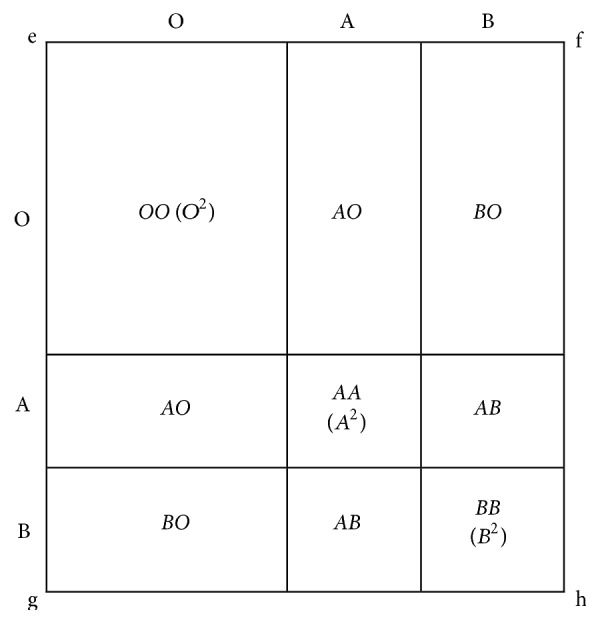
Deducing the gene, genotype, and phenotype frequencies of the ABO blood in a population.

**Table 1:** ABO typing of blood donors at the Lagos University Teaching Hospital University Teaching Hospital.

Blood group	O POS.	O Neg.	A POS.	A Neg.	B POS.	B Neg.	AB POS.	AB Neg.	Total

Number of donors	4679	283	2001	97	1677	94	281	26	**9138**

**%**	51.2	3.1	21.9	1.1	18.4	1.0	3.0	0.3	**100**

POS.: positive.

Neg.: negative.

## Appendices

### A. Arithmetical Calculation of Population Prevalence of ABO Genes from Phenotype Data

From Figure 1, the gene, genotype, and phenotype frequencies of the ABO blood groups can be deduced.

According to Mourant et al. [24], square e-f-g-h in Figure 1 has a breadth and length of unity such that the area of the square is also unity. If along the length and breadth we have genes O, A, and B representing the ABO genes of both parents of a child, the possible genotypes of the offspring are represented in the smaller boxes within the bigger square and these possible genotypes can easily be derived from expansion of binomial (A + B + O)^2^ to give (*A* ^2^ + *B* ^2^ + *O* ^2^ + 2*AB* + 2*AO* + 2*BO*).

From this expansion, we can estimate the population prevalence of different ABO genotypes in a given population if the prevalence of some of the genotypes is known particularly the genotype that can easily be derived from knowing the prevalence of the phenotype:

Phenotype O has genotype* OO* (O =* OO*).
Phenotype A has genotype* AA* +* AO* (A =* AA* +* AO*).
Phenotype B has genotype* BB* +* BO* (B =* BB* +* BO*).
Phenotype AB has genotype* AB* (AB =* AB*).

It is conventional to represent genes A, B, and O with letters* p*,* q,* and *r*, respectively.

Thus, genotype* AA* prevalence is a square of the prevalence of gene A in a given population; that is,

(A.1) $$\begin{array}{c} AA=A^{2}=p^{2}. \end{array}$$

Similarly,

(A.2) $$\begin{array}{c} BB=B^{2}=q^{2}, \end{array}$$

(A.3) $$\begin{array}{c} OO=O^{2}=r^{2}. \end{array}$$

From Figure 1 or the expansion of the binomial (A + B + O)^2^ to give (*A* ^2^ + *B* ^2^ + *O* ^2^ + 2*AB* + 2*AO* + 2*BO*) prevalence of genotype* AO* will be given by

(A.4) $$\begin{array}{c} AO=2AO=2pr. \end{array}$$

Similarly,

(A.5) $$\begin{array}{c} BO=2BO=2qr,\\ AB=2AB=2pq. \end{array}$$

Equations (A.1) to (A.5) shall now be used to calculate the prevalence of the ABO genes in a given population.

A gene occurs in genotypes* AA* and* AO*. Thus prevalence of A gene is given by

(A.6) $$\begin{array}{c} A=\left(AA+AO\right)=\left(p^{2}+2pr\right). \end{array}$$

Similarly,

(A.7) $$\begin{array}{c} B=\left(BB+BO\right)=\left(q^{2}+2qr\right). \end{array}$$

Adding (A.6) and (A.3), we have,

(A.8) A+O=p2+2pr+r2  Hardy  Weinberg  Equation.

Extracting square root of (A.8),

(A.9) $$\begin{array}{c} \sqrt{A+O}=\left(p+r\right). \end{array}$$

Since* p* +* q* + *r* = 1 (i.e., sum of prevalence of A, B, and O genes in a given population),

(A.10) $$\begin{array}{c} q=1-\left(p+r\right)=1-\sqrt{A+O}. \end{array}$$

Similarly,

(A.11) $$\begin{array}{c} p=1-\left(q+r\right)=1-\sqrt{B+O}. \end{array}$$

From (A.3), we can derive that prevalence of gene O that is, *r*, is

(A.12) $$\begin{array}{c} r=\sqrt{O^{2}}. \end{array}$$

Applying the above equations to the prevalence of ABO blood group phenotypes from the record of 9138 blood donors at the Lagos University Teaching Hospital donor clinic we estimate the following.

4962 of 9138 (54.3%) donors were blood group O, 23.0% (2098) were blood group A, 19.4% (1771) were blood group B, and 3.3% (307) were blood group AB.

From (A.11) $p=1-\sqrt{B+O}$ and using the values above,

(A.13) $$\begin{array}{c} p=1-\sqrt{0.194+0.543}=1-\sqrt{0.737}=1-0.8585=0.1415. \end{array}$$

Therefore population frequency for A gene = *p* = 0.1415.

From (A.10), also using the above values,

(A.14) $$\begin{array}{c} q=1-\sqrt{0.23+0.543}=1-\sqrt{0.773}=1-0.8792=0.1208. \end{array}$$

Therefore population frequency for B gene = *q* = 0.1208.

From (A.3), we can say that

(A.15) $$\begin{array}{c} O^{2}=r^{2}=0.543. \end{array}$$

Therefore population frequency for O gene = $\sqrt{O^{2}}=\sqrt{0.543}=0.7369$.

Thus *r* = 0.7369.

#### A.1. Corrected ABO Gene Frequencies

According to Bernstein [25] the sum total of* p*,* q*, and *r* does not usually sum up to 1. The* p*,* q*, and *r* estimates need to be corrected using a quantity *D* which is defined as

(A.16) $$\begin{array}{c} D=1-\left(p+q+r\right). \end{array}$$

From addition above, (*p* +* q* +* r*) = 0.1415 + 0.1208 + 0.7369 = 0.9992. Consider

(A.17) $$\begin{array}{c} D=1-0.9992=0.0008. \end{array}$$

The corrected frequencies for *p*(*p*_*c*_), *q*(*q*_*c*_), and *r*(*r*_*c*_), according to Bernstein, are

(A.18) $$\begin{array}{c} p_{c}=\frac{p}{1-D}=\frac{0.1415}{1-0.0008}=\frac{0.1415}{0.9992}=0.1416,\\ q_{c}=\frac{q}{1-D}=\frac{0.1208}{1-0.0008}=\frac{0.1208}{0.9992}=0.1209,\\ r_{c}=\frac{r}{1-D}=\frac{0.7369}{1-0.0008}=\frac{0.7369}{0.9992}=0.7375. \end{array}$$

Thus the calculated gene frequencies were 0.1416, 0.1209, and 0.7375 for the A, B, and O genes, respectively.

### B. Calculating Likelihood of Scenario Where a Non-Blood-Group-O Child Is Born to a Blood Group O Mother

For ABO hemolytic disease of the newborn to occur, a blood group O woman must be married to a non-group-O man and have an offspring who is non-blood-group-O.

The probability will be the summation of probability of occurrence of blood group genotypes* AA*,* BB*,* AO*,* BO*, and* AB* husband being married to group* OO* genotype wife resulting in an offspring with non-group-O gene.

Using the gene frequencies calculated above, one has the following.

The probability that an* OO* genotype woman will be married to* AA* genotype is the product of frequency of* OO* genotype and frequency of* AA* genotype:

(B.1) $$\begin{array}{c} AA=r^{2}\times p^{2}. \end{array}$$

Therefore,

(B.2) $$\begin{array}{c} AA=0.7375^{2}\times 0.1416^{2}=0.010906. \end{array}$$

Similarly,

(B.3) $$\begin{array}{c} BB=0.7375^{2}\times 0.1209^{2}=0.007950. \end{array}$$

The probability that an* OO* genotype woman will be married to* AO* genotype man will be given by

(B.4) $$\begin{array}{c} AO=r^{2}\times 2pr. \end{array}$$

The probability that the child will inherit the A gene from the father is half. Therefore the probability that an* AO* man and an* OO* woman will have an* AO* child will be given by

(B.5) $$\begin{array}{c} AO=\frac{\left(r^{2}\times 2pr\right)}{2}=r^{2}\times pr,\\ AO=0.7375^{2}\times 0.1416\times 0.7375=0.056800. \end{array}$$

Similarly,

(B.6) $$\begin{array}{c} BO=0.7375^{2}\times 0.1209\times 0.7375=0.048497. \end{array}$$

The probability that* OO* genotype woman will be married to* AB* genotype man and the child will have either A or B gene is given by

(B.7) $$\begin{array}{c} AB=r^{2}\times 2pq,\\ AB=0.7375^{2}\times 2\times 0.1416\times 0.1209=0.018623. \end{array}$$

The sum of all the probabilities = 0.142776.

Therefore, 14.3% of deliveries in Lagos University Teaching Hospital University Teaching Hospital will result in a woman whose blood group is O giving birth to a child who has a non-group-O phenotype.
